# Protective effect of grape seed and skin extract against high-fat diet-induced dyshomeostasis of energetic metabolism in rat lung

**DOI:** 10.1186/s12944-018-0754-0

**Published:** 2018-05-10

**Authors:** Mohamed El Ayed, Safwen Kadri, Maha Mabrouk, Ezzedine Aouani, Salem Elkahoui

**Affiliations:** Bioactive Substance Laboratory, Biotechnology Centre, Technopolis Borj-Cedria, BP-901, 2050 Hammam-Lif, Tunis, Tunisia

**Keywords:** Obesity, High-fat-diet, Lung, Grape seed and skin extract, Oxidative stress, Energy metabolism, Metal ions

## Abstract

**Background:**

Obesity is currently one of the major epidemics of this millennium and affects poeples throughout the world. It causes multiple systemic complications as it significantly interferes with respiratory function.

**Objective:**

We aimed in the present work to study the effect of high fat diet (HFD) on lung oxidative stress and energy metabolism alterations, as well as the putative protection afforded by grape seed and skin extract (GSSE).

**Methods:**

We started by characterizing the GSSE and its composition using gas chromatography coupled to mass spectrometry (GC-MS). We used a rat model of high-fat-diet and we evaluated the effect of GSSE on oxidative stress and energetic disturbances induced by HFD. We analyzed the effect of HFD on lung oxidative status by assessing lipid oxidation level, non-protein thiols (NPSH) and superoxide anion level… We also evaluated the effect of HFD on creatine kinase (CK), malate dehydrogenase (MDH) and mitochondrial complex IV.

**Results:**

HFD induced body weight gain, increased lung weight and lipid content without affecting insulinemia and dropped adiponectemia. HFD also provoked on lung oxidative stress characterized by increased carbonylation (+ 95%; *p* = 0.0045), decreased of NPSH (− 32%; *p* = 0.0291) and inhibition of antioxidant enzyme activities such as glutathione peroxidase (− 25%; *p* = 0.0074). HFD also altered lung intracellular mediators as superoxide anion O^2^¯ (+ 59%; *p* = 0.0027) and increased lung xanthine oxidase activity (+ 27%; *p* = 0.0122). HFD induced copper depletion (− 24%; *p* = 0.0498) and lead (− 51%: *p* = 0.0490) from the lung. Correlatively HFD decreased the copper associated enzyme tyrosinase (− 29%; *p* = 0.0500) and decreased glutamine synthetase activity (− 31%; *p* = 0.0027). HFD altered also lung energy metabolism by increasing CK activity (+ 22%; *p* = 0.0108) and decreasing MDH and mitochondrial complex IV activities (− 28%; *p* = 0.0120, − 31%; *p* = 0.0086 respectively). Importantly all these alterations were efficiently corrected with GSSE treatment.

**Conclusion:**

In conclusion, GSSE has the potential to alleviate the deleterious lipotoxic effect of HFD on lung and it could find potential application in the protection against HFD-induced lung complications.

## Background

In recent decades, the prevalence of obesity has increased dramatically, and it has become the most common metabolic disease worldwide, leading to a global epidemic [1]. Several systemic complications are associated with obesity, some of which lead to severe impairment of organs and tissues. These complications involve mechanical changes caused by the accumulation of adipose tissue and the numerous cytokines produced by adipocytes [2]. However, obesity- induced adipokine dysfunction promotes pulmonary diseases including asthma, chronic obstructive pulmonary disease and pulmonary hypertension. The effects of obesity on the respiratory system have been increasingly studied. The accumulation of fat in the body causes changes in respiratory physiology, with consequent impairment of various lung function parameters [3]. A high-fat diet is known to contribute to obesity. Little is known regarding the effect of a high-fat diet on pulmonary function, despite the dramatic increase in the prevalence of respiratory ailments [4].

Up to now, many bioactive food components like those found in grape have been shown to prevent from a wide array of chronic disorders linked to metabolic syndrome [5]. Grape seed and skin extract (GSSE) is a polyphenol rich mixture [6] commonly used as nutritional supplement [7]. GSSE exerts numerous biological activities and health-promoting properties such as antioxidant [8], lipid lowering [9], and anti-obesity effects [10]. The effect of grape polyphenols on experimentally- induced obesity has been largely approached [11].

In the present work, we studied the potential anti-obesity effect of high dosage GSSE in high-fat-diet (HFD)-induced obesity in rat, with an emphasis on the protection afforded against lipotoxicity-induced lung oxidative stress and energy metabolism alteration. Data mainly showed that obesity induced oxidative stress into lung and altered energy metabolism and that GSSE prevented efficiently from the disturbances elicited upon HFD treatment.

## Materials and methods

### Grape seed and skin extract preparation

Grape seed and skin extract was processed from a grape cultivar (Carignan) of *Vitis vinifera* from northern Tunisia. Seeds were manually separated from skin, air-dried and grounded separately with a coffee grinder (FP 3121Moulinex) until a fine powder was obtained. Both powders were then mixed at 50:50 ratios on a dry weight basis in 10% ethanol (*v*/v) at dark. After vigorous stirring and centrifugation at 10,000 g for 15 min at 4 °C, supernatant containing soluble polyphenols was used. Total phenolic content was determined by the Folin- Ciocalteau colorimetric method [12]. Additionally, the Dewanto et al. [13] and Sun et al. [14] methods have been used for flavonoid and condensed tannin determination respectively. GSSE composition was established using gas chromatography coupled with mass spectrometry (GC-MS) according to the procedures described by Erbing et al. [15].

Briefly, hydrolysis of GSSE was performed by treatment with 2 M trifluoroacetic at 120 °C for 2 h and hydrolysates were converted into 100 μL dichloromethane. Extracts were performed under diminished pressure under a stream of helium N60. For GC, a Agilent 7890A GC instrument. GC-MS (EI, 70 eV) was performed on a Agilent 5975C inert GC-MSD instrument with triple-axis detector. GSSE were analyzed on HP-5 capillary column (30 m × 0.25 mm coated with 5% phenylmethylpolysiloxane × 0.25 μm) using the temperature program150°C (1 min); 180 °C at 1 °C/min; 250 °C at 2 °C/min; 300 °C at 15 °C/min and at 300 °C (10 min).

### Animals diets and experimental design

Twenty-four male Wistar rats (190–200 g) from Pasteur Institute Tunis were used in these experiments in accordance with the National Ethic Committee of Tunis University for use and care of animals in conformity with the NIH recommendations [16]. They were maintained in animal facility at a controlled temperature (22 ± 2 °C), a 12 h (light- dark) cycle, and divided into four groups of six animals each that were daily treated as follows:

Group1: SD: Rats fed Standard Diet (SD) and administered with 10% ethanol during 7 weeks.

Group 2: GSSE: Rats fed SD and treated with 4 g/kg bw GSSE during 7 weeks.

Group 3: HFD: Rats fed High Fat Diet (HFD) and administered with 10% ethanol during7 weeks.

Group 4: HFD + GSSE: Rats fed HFD and treated with 4 g/kg bw GSSE during 7 weeks.

SD for rodent in pellet was purchased from BADR Bizerte (Tunisia) and consisted of 3% fat, 40% carbohydrate, 14% protein. HFD was prepared by soaking commercial food pellets into warmed (100 °C) and liquefied fat (peri-renal) of animal origin (sheep) for 15 min and allowed to dry at room temperature. HFD contained 28% fat, 32% carbohydrate, and 12% protein [17].

At the end of the treatment period, rats were anesthetized with urethane (40 mg. mL¯^1^), sacrificed by decapitation. Blood was collected using heparin as anticoagulant.

### Tissue homogenization

Lungs were isolated, weighed, and homogenized in phosphate buffered saline (PBS; pH = 7.4; 50 mM) with an ultrathurax homogenizator. After centrifugation (10 min at 10000 g, 4 °C), supernatant was frozen in liquid nitrogen and stored at 80 °C until assays. Body weight evolution was determined, lipids were extracted from whole lungs according to Folch et al. [18], and lung lipids conent was estimated.

### Plasma analyses

Plasma insulin was measured using the ultrasensitive rat insulin ELISA Kit (Alpco Diagnostics) and adiponectin was measured using the Assay Max rat adiponectin ELISA Kit (Assay paro) [16].

### Biochemical analyses

#### Lung carbonylation

Oxidative damage to proteins was evaluated by quantifying protein carbonylation in lung homogenates according to Levine et al. [19]. Briefly, after proteins precipitation with 20% TCA and centrifugation at 11000 g for 3 min at 4 °C, pellet was dissolved in 10 mM dinitrophenylhydrazine (DNPH) containing phosphate buffer. After 3 washings with ethanol-ethylacetate (1:1), the last pellet obtained was dissolved in 20 mM potassium phosphate pH 2.3 containing 6 M guanidine HCl and absorbance measured at 366 nm using the molar extinction coefficient of 22,000 M¯^1^.cm¯^1^ and result expressed as mmol carbonyl protein/mg protein. Total soluble proteins were determined according to the biuret method [20]. Non-protein thiols (NPSH) were determined according to Ellman [21].

#### Antioxidant glutathione peroxidase activity

Lung homogenate were also used to evaluate endogenous antioxidant enzyme activity as glutathione peroxidase (GPx; EC 1.11.1.9) according to Charradi et al. [22]. Briefly, lung homogenate (2 mg protein) were mixed with phosphate buffer 100 mM pH = 7.4 containing 4 mmol GSH and 5 mmol H2O2 in 1 mL final volume. The mixture was incubated at 37 °C for 1 min; then, 0.5 mL TCA (5%, *w*/*v*) was added to stop the reaction. After centrifugation at 1.500 g for 5 min, 0.2 mL supernatant was mixed with phosphate buffer 100 mmol pH = 7.4 containing 10 mmol 2-nitrobenzoc acid (DTNB). GPx activity was measured at 412 nm.

#### ROS measurement

Lung superoxide radical was determined according to Marklund and Marklund. [23] with slight modifications. Briefly after incubation of brain homogenate in Tris- HCl buffer pH = 8·2 at 25 °C for 10 min, pyrogallol was added to the mixture and the incubation pursued at 25 °C for four-minutes. The reaction was terminated by the addition of HCl and absorbance measured at 420 nm against the blank.

#### Xanthine oxidase activity

Lung xanthine oxidase (XO) activity (EC.1.17.3.2) was determined according to Avis et al. [24] The principle of this method is as follows: In the presence of xanthine, XO produced uric acid and absorbance measured at 295 nm using the molar extinction coefficient of the acid uric = 9500 Mˉ^1^.cmˉ^1^.

#### Metals ions measurements and some enzymes activities

Tissue samples were also washed in nitric acid (15. 5 mol. L¯^1^), diluted, and filtered for copper and plumb measurements by atomic absorption spectroscopy. Tyrosinase activity (EC. 1.14.18.1) a copper (Cu^2+^) dependent enzyme was determined using L- tyrosine as substrate in 50 mM sodium phosphate buffer pH = 6.5 at 25 °C [25]. Glutamine synthetase (GS) activity (EC. 6.3.1.2) a Manganese (Mn) containing enzyme was determined according to Santoro et al. [26]. Briefly, lung homogenate was added to assay mixture containing 50 mM imidazole, 25 mM arsenic, 0.16 mM ADP, 50 mM L-glutamine, 25 mM hydroxylamine and 2 mM MnCl2. Incubation was carried out at 37 °C for one-hour and the reaction stopped by addition of two volumes of a ferric chloride solution containing 2.42% ferric chloride, 1.45% trichloroacetic acid (TCA) and 1.82% HCl. After centrifugation at 10000 g for 10 min absorbance of the supernatant was read at 540 nm.

#### Mitochondrial isolation

Lung tissue was homogenized in mitochondrial isolation buffer containing 70 mm sucrose, 210 mm mannitol, 5 mm Tris HCl, 1 mm EDTA; pH = 7.4 and suspensions were centrifuged at 800 g, 4 °C, for 10 min. The supernatant fluids were centrifuged at 13000 g, 4 °C, for 10 min, and the pellets were washed with mitochondrial isolation buffer and centrifuged at 13000 g, 4 °C, for 10 min to obtain the crude mitochondrial fraction [27].

#### Mitochondrial enzymes activities

##### Creatine kinase activity

Creatine kinase (CK) activity (EC.2.7.3.2) was determined from mitochondrial fraction using a commercial kit from Biomaghreb, Tunisia. Briefly, CK was reactivated by N-acetylcysteine and CK activity was measured following the decrease in NADH which absorbed at 340 nm and expressed as follows: ΔDO/min * 4130.

##### Malate dehydrogenase activity

The activity of malate dehydrogenase (MDH) (EC.1.1.1.37) was measured as described previously by Kitto [28] with slid modification. Aliquots (20 μg protein) were transferred into a medium containing 10 mM rotenone, 0.2% Triton X-100, 0.15 mM NAD, and 100 mM potassium phosphate buffer, pH 7.4, at 37 °C. The reaction was started by addition of 0.33 mM malate. The absorbance was monitored at 340 nm.

##### Activity of mitochondrial complex-IV

Mitochondrial complex-IV (Cytochrome c oxidase, EC.1.9.3.1) activity was measured spectrophotometrically according to the method of Ragan et al. [29] as modified by Desai et al.(28) and it measured as the rate of oxidation of reduced cytochrome c by mitochondria at 30 °C. The decrease in reduced cytochrome c was monitored at 550 nm.

### Statistical analysis

Results are expressed by mean ± SEM. Significance between groups was evaluated using Student’s *t*-test. For multiple comparisons, one- way analysis of variance (ANOVA) and *p* < 0.05 was considered significant: (*) for HFD vs. SD, and (§) for HFD + GSSE vs. HFD. (**) and (§§) for *p* < 0.01.

## Results

### Grape seed and skin extract composition

Phenolics levels found in GSSE from Carignan cultivar prepared in H2O, 10% Ethanol and 100% Ethanol were given in Table 1. Total phenolics, flavonoids and condensed tannins were slightly higher in the 100% ethanol extract than 10% ethanol.

**Table 1:** Phenolic levels in GSSE

Phenolics	H2O	10% ethanol	100 ethanol
Total phenolics (mg EGA/ g Extract)	0.68 ± 0.02	0.71 ± 0.01	0.83 ± 0.02
Total flavonoids (mg EC/g Extract)	0.16 ± 0.01	0.19 ± 0.01	0.20 ± 0.01
Condensed Tannins (mg EC/g Extract)	0.02 ± 0.01	0.06 ± 0.02	0.14 ± 0.04

Fifteen phenolic compounds were identified in 10% GSSE using GC-MS (Table 2). Among these compounds, epicatechin (35.21%) and catechin (34.41%) were the most abundant.

**Table 2:** GC-MS data of phenolic compounds found in Carignan GSSE

Compounds	Relative abundance (%)
Tyrosol	1.03
Syringaldehyde	0.23
Vanillin	0.14
Protocatechin acid	2.2
7, 9-Di-tert-butyl-1-oxaspiro (4, 5) deca-6, 9-diene-2, 8-Dione	1.21
p- Coumaric acid	0.54
Ethyl ester gallic acid	1.73
Gallic acid	12.84
Ferulic acid	0.27
Squalen	1.2
Epicatechin	35.21
Catechin	34.41
Flavan-3-ol	2.25
Quercetin	1.01
Quercetin derivatives	5.73

### Biometric parameters

**Fig. 1:**
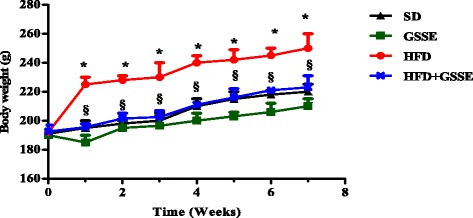
Effect of HFD and GSSE on body weight evolution. Rats were fed a SD or HFD during 7 weeks and daily treated with GSSE (4 g/kg). Results are expressed as mean ± SEM (*N* = 6). *p* < 0.05 was considered significant: (*) for HFD vs. SD, and (§) for HFD + GSSE vs. HFD. (**) and (§§) for *p* < 0.01

**Fig. 2:**
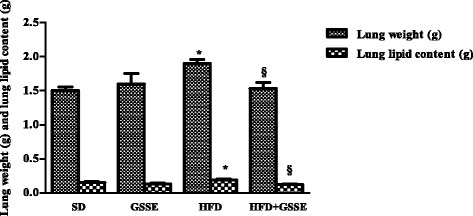
Effect of HFD and GSSE on lung weight and lung lipids content. Results are expressed as mean ± SEM (*N* = 6). *p* < 0.05 was considered significant: (*) for HFD vs. SD, and (§) for HFD + GSSE vs. HFD. (**) and (§§) for *p* < 0.01

We reported in Fig. 1 the effect of HFD and GSSE on body weight evolution over time. Data showed that after 7 weeks long fat treatment, the animals become obese and that treatment with high dosage GSSE (4 g/kg) prevented efficiently bw gain till control SD animals. HFD resulted in a marked increase in lung weight (26%) and lung lipid content (32%). Treatment with GSSE counteracted all HFD-induced disturbances (Fig. 2). HFD was not diabetogenic as it did not affect insulinemia but was obesogenic as indicated by decreased adiponectinemia (Table 3).

**Table 3:** Effect of HFD and GSSE on plasma insulin and adiponectin

	SD	GSSE	HFD	HFD + GSSE
Plasmainsulin(ng/mL)	1.50 ± 0.00	1.75 ± 0.01	1.25 ± 0.01	1.27 ± 0.01
Plasmaadiponectin(ng/mL)	6.20 ± 0.02	6.30 ± 0.01	2.75 ± 0.04**	4.95 ± 0.02§

Results are expressed as mean ± SEM (*N* = 6). *p* < 0.05 was considered significant: (*) for HFD vs. SD, and (§) for HFD + GSSE vs. HFD. (**) and (§§) for *p* < 0.01

### Lung oxidative stress status

#### Protein carbonylation

**Fig. 3:**
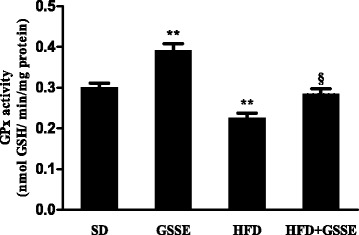
Effect of HFD and GSSE on GPx activity. Results are expressed as mean ± SEM (*N* = 6). *p* < 0.05 was considered significant: (*) for HFD vs. SD, and (§) for HFD + GSSE vs. HFD. (**) and (§§) for *p* < 0.01

We evaluated the effect of HFD on lung protein carbonylation and NPSH content. HFD increased lung protein carbonylation (Fig. 3) by 95% (*P* = 0.0045) and decreased NPSH content (Table 4) by 32% (*P* = 0.0291). GSSE counteracted efficiently the HFD-induced disturbances until control levels.

**Table 4:** Effect of HFD and GSSE on lung protein carbonylation and NSPH

	SD	GSSE	HFD	HFD + GSSE
Carbonyl protein (mmol carbonylated protein/mg protein)	0.0116 ± 0.001249	0.008 ± 0.001528	0.02267 ± 0.001453^**^	0.01667 ± 0.0008819^§^
NPSH (mmol/mg protein)	12.13 ± 0.7535	14.63 ± 0.696	8.233 ± 0.8969^*^	11.73 ± 0.5608^§^

Results are expressed as mean ± SEM (*N* = 6). *p* < 0.05 was considered significant: (*) for HFD vs. SD, and (§) for HFD + GSSE vs. HFD. (**) and (§§) for *p* < 0.01

#### Antioxidant enzyme GPx activity

Furthermore, HFD decreased the antioxidant enzyme GPx activity (Fig. 3) by 25% (*P* = 0.0074). GSSE backed HFD-induced drop in GPx to near control and GSSE on its own increased GPx activity by 30%.

#### ROS measurement

**Fig. 4:**
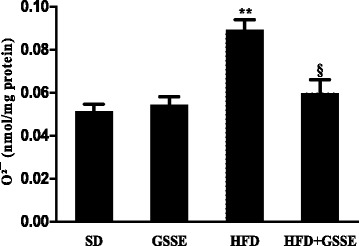
Effect of HFD and GSSE on lung O^2^¯ activity. Results are expressed as mean ± SEM (*N* = 6). *p* < 0.05 was considered significant: (*) for HFD vs. SD, and (§) for HFD + GSSE vs. HFD. (**) and (§§) for *p* < 0.01

We further asked whether HFD affect O^2^¯ lung level. Data clearly showed that HFD increased O^2^¯ (Fig. 4) by 59% (*P* = 0.0027) and that GSSE treatment backed them to near control level.

#### Xanthine oxidase activity

**Fig. 5:**
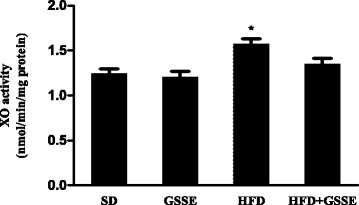
Effect of HFD and GSSE on xanthine oxidase activity (XO). Results are expressed as mean ± SEM (*N* = 6). *p* < 0.05 was considered significant: (*) for HFD vs. SD, and (§) for HFD + GSSE vs. HFD. (**) and (§§) for *p* < 0.01

We then sought to determine the impact of HFD on xanthine oxidase activity (XO), which is associated with the production of free radicals. HFD increased lung XO activity (Fig. 5) by 27% (*P* = 0.0122). GSSE counteracted the impact of HFD as it restored the enzyme activity to near control level.

#### Metals ions measurement and some associated enzymes

HFD induced depletion of Cu^2^+ by 24% (*P* = 0.0498) and decreased Pb by (51%) (*P* = 0.0490). HFD decreased the copper dependent enzyme tyrosinase activity by 29% (*P* = 0.0500) and increased the -manganese depending- glutamine synthetase activity (GS) by 31% (*P* = 0.0027) (Table 5). GSSE efficiently counteracted the effect of HFD on metal ions and the associated enzyme activities disturbances.

**Table 5:** Effect of HFD and GSSE on Copper; Lead; tyrosinase activity and glutamine synthetase activity

	SD	GSSE	HFD	HFD + GSSE
Cu2+ (mg/L)	286 ± 22.72	359.4 ± 33.32	216.5 ± 10.45^*^	308.2 ± 13.6^§^
Pb (μg/ L)	1673 ± 266.8	1637 ± 234.4	813.7 ± 152.2^*^	1575 ± 173^§^
Tyrosinase activity (U/ mg protein)	0.1383 ± 0.01239	0.1423 ± 0.006984	0.09833 ± 0.007356^*^	0.1393 ± 0.005783
Glutamine synthetase activity (nmol/min/mg protein)	1.947 ± 0.04842	1.913 ± 0.1618	1.353 ± 0.1179^*^	1.667 ± 0.1168^§^

Results are expressed as mean ± SEM (*N* = 6). *p* < 0.05 was considered significant: (*) for HFD vs. SD, and (§) for HFD + GSSE vs. HFD. (**) and (§§) for *p* < 0.01

### Lung energy metabolism status

#### Creatine kinase activity

**Fig. 6:**
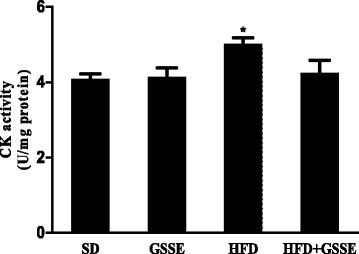
Effect of HFD and GSSE on creatine kinase activity (CK). Results are expressed as mean ± SEM (*N* = 6). *p* < 0.05 was considered significant: (*) for HFD vs. SD, and (§) for HFD + GSSE vs. HFD. (**) and (§§) for *p* < 0.01

HFD affected also on creatine kinase activity (CK) which plays a key role on the energetic metabolism. HFD highly increased lung CK activity (Fig. 6) by 23% (*P* = 0.0108), while GSSE reversed these HFD induced disturbance to control level.

#### Krebs cycle enzyme activities

**Fig. 7:**
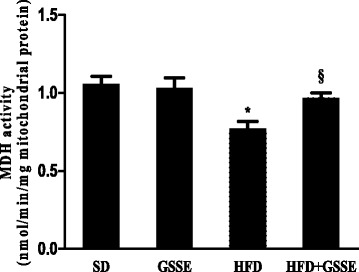
Effect of HFD and GSSE on malate dehydrogenase activity (MDH). Results are expressed as mean ± SEM (*N* = 6). *p* < 0.05 was considered significant: (*) for HFD vs. SD, and (§) for HFD + GSSE vs. HFD. (**) and (§§) for *p* < 0.01

We interested next to determine the MDH activity, which is an enzyme of Krebs cycle. Data showed that HFD decreased MDH activity (Fig. 7) by 27% (*P* = 0.0120). GSSE backed them to near control level.

#### Activity of mitochondrial respiratory chain complex IV

**Fig. 8:**
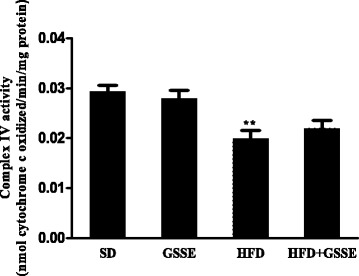
Effect of HFD and GSSE on cytochrome c oxidase (complex IV). Results are expressed as mean ± SEM (*N* = 6). *p* < 0.05 was considered significant: (*) for HFD vs. SD, and (§) for HFD + GSSE vs. HFD. (**) and (§§) for *p* < 0.01

Finally, we assessed the effect of HFD activity on mitochondrial respiratory chain complex IV. HFD highly decreased the enzyme activity (Fig. 8) by 31% (*P* = 0.0086). GSSE corrected this activity to near control level.

## Discussion

The present paper deals with the effect of HFD on rat lung as well as the putative protection offered by GSSE.

HFD induced lipotoxicity was assessed by altered body weight, lung weight and lipid content. However, HFD induced body weight gain, provoked lipotoxicity without affecting insulinemia and decreased adiponectinemia. HFD-induced lipotoxicity also generated an oxidative stress into the lungs. Thus, HFD increased lung carbonylation, decreased non protein thiols and altered antioxidant enzyme activities as GPx. Our data fully agree with several previous works in the field [16, 30–32], who showed that HFD provoked an oxidative stress in the heart, liver, brain, kidney and muscles and particularly with the recent work of Charradi et al. [32] who showed that HFD increased carbonylation and dropped NPSH in brain. Moreover, we showed that HFD depresses GPx activity. Furthermore, our results showed that HFD also elicited an increase in harmful ROS, as superoxide. The increase in superoxide likely results from the inhibition in SOD activity that occurs upon HFD- induced lipotoxicity. HFD induced lung oxidative stress was also characterized by elevated activity of xanthine oxidase, which is associated with the production of free radicals and formation of uric acid. Our data are in line with previous studies [33–35] who demonstrated that elevated XO activity and uric acid levels are known to correlate with obesity. Similarly, Kelley et al. [32] showed that HFD increasing lung XO activity.

The effect of HFD on lung metals ions level showed that it induced the depletion of Cu^2^+ and pb^2+^. These data were further supported by the similar variation that occurred in copper dependent enzyme such as tyrosinase as well as on glutamine synthetase activity. Whereas we found that HFD induced a clear drop in GS activity, which play a key role in glutamine homeostasis. However and during physiological stress, the lung should increase production of the amino acid glutamine to maintain glutamine homeostasis [36]. Noteworthy that in our current study, HFD has affect copper and their associated enzyme tyrosinase. To the best of our knowledge, our data are the first to link HFD induced oxidative stress in the lung with the dyshomeostasis of copper and plumb i.e. depletion of copper and a drastic depletion of plumb.

Another relevant effect of HFD is its ability to affect energy metabolism [37–39]. The overall role of intermediary metabolism in cell homeostasis is primarily to generate the requisite energy for cellular functions and to provide the small molecular weight substrates for biosynthetic processes. In lung cells as in most tissues, a variety of substrates derived from protein, lipid and carbohydrate precursors can enter the cellular pool of intermediary metabolites. Thus, lung tissue can oxidize to different degrees glucose, fatty acids, amino acids and lactate [39, 40]. However, the rate of glucose oxidation is greatest [41]. The adenosine triphosphate (ATP) content of the normal perfused lung is approximately 2 to 2.5 μmole/g lung weight. This is similar to ATP values for rat brain, liver and kidney. However, our data showed that HFD increased the lung CK activity, which is the central regulatory enzyme of energy metabolism.

In addition, HFD altered the Krebs cycle enzymes activity by decreasing MDH activity.

(− 27%) which is a key enzyme and affected the electron transport chain by increasing complex IV enzyme activity (− 31%). This is can be partially explained by the increased resistance imposed by the presence of excess fatty tissue on the chest and abdomen, which causes mechanical disadvantage to these muscles and production of energy will be increased.

The current finding that GSSE can be benefic in the context of obesity induced pulmonary complication is novel. Although the mechanism underlying this effect are not completely clear. The use of nutraceutical in dyslipidemia has been studied previously [42]. Many molecules has proved beneficial effects on dyslipidemia such as resveratrol [43], water-insoluble fish proteins from Alaska Pollock [44] and curcumin [45]. However mechanisms are not fully understood and might implicate several pathways. In the current study we present evidence that increased stress oxidative, energy metabolism deregulation are present in our model, and undoubtedly, the most relevant result drawn is the protection offered by GSSE against HFD-induced lung lipotoxicity, oxidative stress and dysregulation energy metabolism. We do not yet know which kind of GSSE containing-polyphenol is at the basis of such protection. Our present data are in favor of a synergism between the numerous GSSE-containing polyphenols rather than the specific effect of a single compound.

In conclusion, we have demonstrated that GSSE has beneficial effects in reversing almost all the negative effects of HFD on lung. The research work provides evidence that GSSE supplementation in rats with obesity induced by HFD protected against oxidative stress and energy metabolism dysregulation in the lung. Finally, the effects of GSSE on HFD-induced rats indicate that GSSE possesses anti-obesity effects and may curtail obesity-related symptoms, including pulmonary complication like asthma [46] and raise the possibility of new applications. Such results should emphasizes the use of GSSE supplementation in everyday life of over-weighted persons and opens perspectives for clinical trials.

## Abbreviations

ATP: Adenosine triphosphate
CK: Creatine kinase
GC–MS: Gaz chromatography-mass spectrometry
GPx: Glutathione peroxidase
GS: Glutamine synthase
GSSE: Grape seed and skin extract
HFD: High fat diet
MDH: Malate dehydrogenase
SD: Standard diet
XO: Xanthine oxidase
